# Contamination of perfusion fluid and its impact on kidney transplantation: an observational study from a single Brazilian center

**DOI:** 10.1590/2175-8239-JBN-2025-0037en

**Published:** 2025-08-08

**Authors:** Letícia Aguirre Mantoani, Letícia Segura Graciani, Raquel Hernandez Bertine, João Fernando Picollo Oliveira

**Affiliations:** 1 Faculdade de Medicina de São José do Rio Preto, São José do Rio Preto, SP, Brazil.; 2 Faculdade de Medicina de São José do Rio Preto, Departamento de Nefrologia, São José do Rio Preto, SP, Brazil.

**Keywords:** Culture, Infections, Perfusion, Transplantation

## Abstract

**Introduction:**

Infections represent a major cause of morbidity and mortality in kidney transplant recipients. Preservation fluid (PF) contamination is considered a potential infectious source; however, its clinical relevance remains controversial.

**Aim:**

To evaluate whether PF contamination acts as a source of early post-transplant infections (within 30 days) and its association with acute rejection, graft loss, and mortality within 90 days.

**Methods:**

This was a retrospective, observational, and descriptive study based on medical records of patients aged ≥18 years who underwent kidney transplantation between January 2021 and December 2023. Collected variables included demographic, clinical, and post-transplant outcome data.

**Results:**

Among 246 recipients with available PF culture data, 27.6% (68/246) presented with PF contamination. Gram-positive cocci accounted for 64.7% of isolates, Gram-negative bacilli, for 35.3%, and fungi, for 2.9%. Coagulase-negative staphylococci (CoNS) were the most frequent isolate (36.8%). Microbiological concordance between PF isolates and pathogens responsible for early infection were observed in 13.23% (9/68) of cases, with *Klebsiella pneumoniae* being the predominant pathogen (66.6%). Although the infection rate was higher among patients with positive PF cultures (72%) compared to those with negative cultures (64%), this difference was not statistically significant (p = 0.2992). No significant associations were found with mortality (p = 1.000), graft loss (p = 0.8199), or acute rejection (p = 0.5635).

**Conclusion:**

PF contamination was frequent and may contribute to early post-transplant infections, reinforcing the importance of microbiological surveillance and preventive strategies.

## Introduction

Kidney transplantation is considered the treatment of choice for many end-stage renal diseases. Although essential for maintaining organ viability, perfusion fluid (PF) may serve as a vector for microbial transmission during organ procurement and transplantation.

Organ preservation is typically achieved using PF, which is applied immediately after organ removal from the donor to prolong its viability outside the human body. During this process, the graft is particularly susceptible to contamination by pathogens. Infection is one of the leading causes of morbidity and mortality following a transplant^1^, and may originate from microorganisms already present in the donor, which can be detected in the perfusion fluid.

A French study reported that approximately one in five PF samples from kidney transplants yielded positive cultures^2^, with a higher incidence among deceased donors. Contributing factors to PF contamination may include procedural breaches during retrieval, transport, or back-table preparation prior to implantation^3^. A systematic review and meta-analysis published in 2018^4^, based on 17 previous studies, reported an overall PF contamination rate of 37% and an associated infection rate of 10%.

This study aimed to determine the prevalence of PF contamination in kidney transplants performed at the Hospital de Base in São José do Rio Preto and to investigate its potential association with early post-transplant infections, acute rejection, graft loss, and short-term mortality.

## Methods

### Study Design and Participant

This is a retrospective, observational, and descriptive study approved by the Ethics Committee of the Faculty of Medicine of São José do Rio Preto (FAMERP) through Plataforma Brasil (approval no. 6.561.413; CAAE no. 60072922.3.0000.5415). All data were collected from the hospital’s electronic medical records.

Inclusion criteria were patients aged 18 years or older who underwent kidney transplantation at the Hospital de Base de São José do Rio Preto between January 2021 and December 2023. Exclusion criteria consisted of patients under 18 years of age or those without PF culture data. A total of 282 kidney transplant recipients were identified during the study period, but 271 were aged 18 years or older and 246 had available perfusion fluid culture data. Thus, 246 patients met all the inclusion criteria.

### Group Stratification for Analysi

For comparative analysis purposes, the 246 included recipients were initially divided into two groups based on the culture results of the PF: PF-positive group (n = 68) and PF-negative group (n = 178). The PF-positive group was further subdivided into recipients with and without early infection: PF-positive with early infection (n = 49) and PF-positive without early infection (n = 19), based on the presence or absence of documented clinical infection within the first 30 days post-transplant. This subdivision enabled a more precise evaluation of the impact of microbial contamination of the PF on early clinical outcomes. Statistical analyses were performed to compare outcomes between PF-positive and PF-negative groups, as well as between PF-positive recipients with and without documented early infections.

### Primary and Secondary Outcome

The primary outcome was incidence of infection associated with PF positivity. Secondary outcomes included prevalence of positive PF cultures, incidence of early infections (within 30 days post-transplant), and occurrence of graft loss, acute rejection, or death within 90 days post-transplant.

### Data Collection

Collected variables included patient age, sex, underlying kidney disease, antibiotic prophylaxis, immunosuppressive regimen, mean length of hospitalization, presence and site of early infection, isolated microorganisms, and graft loss, acute rejection, or death. Data were anonymized and stored in a secure database.

### Definition

PF contamination was defined as the presence of any microbial growth in the culture of the PF, irrespective of clinical symptoms or infection. A case was considered to have microbiological concordance when the same pathogen isolated from the PF was later identified in the recipient’s infection site. Infection was defined according to the Centers for Disease Control and Prevention (CDC) and the National Healthcare Safety Network (NHSN)^5^. Ninety-day mortality was defined as death from any cause within the first 90 days after transplantation.

### Clinical Protocol

All recipients received intraoperative prophylaxis with cefazolin sodium (1 g), continued for at least three days postoperatively. Sulfamethoxazole-trimethoprim (400 mg + 80 mg) was administered starting at surgery and maintained for at least 90 days. Some patients received meropenem (500 mg/day) and/or vancomycin (15 mg/kg/day), adjusted according to serum vancomycin levels, as a continuation of the antibiotic therapy initiated in the donor. Immunosuppressive induction therapy was performed with either basiliximab or thymoglobulin, based on clinical judgment. Maintenance immunosuppression consisted of tacrolimus, mycophenolate sodium, and prednisone, with individualized dose adjustments based on each patient’s immunological risk profile.

### Statistical Analysi

Statistical analysis was performed using SPSS for Windows, version 23.0 (IBM Corp., Armonk, NY, USA) and GraphPad Instat, version 3.10 (GraphPad Software, San Diego, CA, USA). Categorical variables were presented as absolute frequencies and percentages, and comparisons were made using the chi-square test. Continuous variables were expressed as means with standard deviations or as medians with interquartile ranges (IQR), depending on data distribution. Group comparisons for non-parametric variables were made using the Mann-Whitney U test. All statistical tests were two-tailed, and a p-value of < 0.05 was considered statistically significant.

## Results

Of the 282 kidney transplant recipients during the study period, 271 were aged 18 years or older, and 246 had available PF culture data. Therefore, 36 patients were excluded. The mean age was similar between groups: 46.96 ± 13.74 years in the PF-positive group and 47.46 ± 13.07 years in the PF-negative group (p = 0.7261). The proportion of male patients was also comparable: 64.7% (44/68) in the PF-positive group and 65.7% (117/178) in the PF-negative group (p = 0.999).

All patients received standard antibiotic prophylaxis with cefazolin and sulfamethoxazole-trimethoprim. In a subgroup of patients, meropenem was administered in 2.43% (6/246) of recipients and/or vancomycin in 1.62% (4/246). Most patients received induction immunosuppression with basiliximab (78%; 192/246), while 21.9% (54/246) were treated with thymoglobulin.

Table 1 presents a comparison between PF-positive and PF-negative recipients (n = 246), regardless of infection status.

**Table 1:** Clinical characteristics and outcomes of kidney transplant recipients stratified by pf culture results

Characteristics	Negative PF culture (N = 178)	Positive PF culture (N = 68)	p-value
Sex, male – n (%)	117 (65.73%)	44 (64.70%)	0.999
Age – mean ± SD (years)	47.46 ± 13.07	46.96 ± 13.74	0.7261
Etiology of kidney failure – n (%)			
Diabetes mellitus	12 (6.74%)	10 (14.70%)	0.0877
Hypertension	40 (22.47%)	19 (27.94%)	0.4644
Glomerulonephritis	50 (28.09%)	32 (47.06%)	0.0076
Other causes	76 (42.70%)	7 (10.29%)	< 0.0001
Postoperative metrics			
Hospital stay – median (IQR), days	15.0 (11.0–22.0)	14.0 (10.0–20.0)	0.499
Cold ischemia time – median (IQR), h	23.0 (19.38–27.0)	20.07 (17.73–26.42)	0.2109
Infections – n (%)			
Any infection	114 (64.0%)	49 (72.0%)	0.2992
Urinary tract infection	97 (54.49%)	47 (69.11%)	0.0527
Bloodstream infection	11 (6.17%)	3 (4.41%)	0.8199
Catheter tip infection	23 (12.92%)	6 (8.82%)	0.5027
Secondary outcomes – n (%)			
Acute rejection	8 (4.49%)	5 (7.35%)	0.5635
Graft loss	11 (6.17%)	3 (4.41%)	0.8199
Death	11 (6.17%)	4 (5.88%)	1.000

Note – Values are presented as mean ± standard deviation, median (interquartile range), or n (%), as appropriate. p-values obtained using chi-square or Mann-Whitney U test, as applicable.

Among the 246 recipients included, 27.6% (68/246) had positive PF cultures. A total of 70 microorganisms were isolated from 68 positive preservation fluid samples, including two polymicrobial cultures. These two samples each contained *Acinetobacter baumannii* in association with either *Klebsiella pneumoniae* or *Enterococcus faecalis*. Of the isolates, 64.7% (44/68) were Gram-positive cocci, 35.3% (24/68) Gram-negative bacilli, and 2.9% (2/68) fungi. The most frequent isolates were coagulase-negative Staphylococcus (CoNS), found in 36.8% (25/68) of the samples. ESKAPE pathogens (*Enterococcus faecium*, *Staphylococcus aureus*, *Klebsiella pneumoniae*, *Acinetobacter baumannii*, *Pseudomonas aeruginosa*, and *Enterobacter spp.*) accounted for 36.8% (25/68) of all isolates.

Table 2 presents the distribution of microorganisms isolated from PF samples (N = 68).

**Table 2:** Distribution of microorganisms isolated from preservation fluid samples (N = 68)

Microorganisms	No. (percent)
Gram-positive coccus	
CoNS	25 (36.76%)
*Enterococcus faecalis*	7 (10.29%)
*Corynebacterium sp*	3 (4.41%)
*Staphylococcus aureus*	1 (1.47%)
Others	8 (11.7%)
Gram-negative bacilli	
*Klebsiella pneumoniae*	10 (14.70%)
*Enterobacter cloacae*	5 (7.35%)
*Pseudomonas aeruginosa*	4 (5.88%)
*Acinetobacter baumannii*	2 (2.94%)
Others	3 (4.41%)
Fungus	2 (2.94%)

Abbreviations – CoNS: coagulase negative staphylococcus (*Staphylococcus epidermidis*, *Staphylococcus haemolyticus*, *Staphylococcus hominis*, *Staphylococcus lugdunensis*, *Staphylococcus capitis*, *Staphylococcus warneri*).

Infection occurred in 72% of patients with positive PF cultures versus 64% with negative cultures (p = 0.2992), showing no statistically significant association. Infections related to central venous catheters (p = 0.5027), bloodstream infections (p = 0.8199), and urinary tract infections (p = 0.0527) were not statistically significant, although the latter approached statistical significance. Nine cases of urinary tract infection caused by *Klebsiella pneumoniae* were identified, six of which were associated with PF contamination.

Other secondary outcomes such as death (p = 1.000), graft loss (p = 0.8199), and acute rejection (p = 0.5635) also did not reach statistical significance. Likewise, continuous variables, including length of hospitalization (p = 0.499), cold ischemia time (p = 0.2109), and recipient age (p = 0.999) were not associated with PF culture results. In contrast, a statistically significant association was identified between the underlying cause of end-stage renal disease and PF culture status. Nontraditional causes (excluding diabetes, hypertension, and glomerulonephritis) were more common among PF-negative patients (42.7% vs. 10.3%; p < 0.0001), suggesting a higher prevalence of chronic comorbidities among those with PF-positive cultures.

Table 3 shows microorganisms isolated from the 49 PF-positive recipients who developed early infections.

**Table 3:** Microorganisms isolated from pf-positive recipients with early infections (N = 49)

Microorganisms	Urinary tract infection	Catheter tip infection	Bloodstream infection
CoNS	21	6	2
*Enterococcus faecalis*	3	1	0
*Corynebacterium sp*	1	0	0
Other Gram-positive cocci	2	1	0
*Klebsiella pneumoniae*	**9**	0	0
*Enterobacter cloacae*	3	0	1
Other Gram-negative bacilli	3	0	0
Fungi	2	0	0
Total	44	8	3

Abbreviations – CoNS: coagulase negative staphylococcus (*Staphylococcus epidermidis*, *Staphylococcus haemolyticus*, *Staphylococcus hominis*, *Staphylococcus lugdunensis*, *Staphylococcus capitis*, *Staphylococcus warneri*). Note – This table includes only microorganisms from PF-positive recipients who developed early infections (n = 49). Two polymicrobial PF cultures yielded 70 isolates from 68 samples.

A microbiological match between organisms isolated from PF and those causing early infections (within 30 days) was observed in 13.23% of cases (9/68), with *Klebsiella pneumoniae* being the predominant pathogen, identified in 66.6% (6/9) of these cases.

Among the 68 recipients with positive PF cultures, 72% (49/68) developed early infection. Urinary tract infection was the most frequent type (93.88%; 46/49), followed by central venous catheter-associated infection (12.24%) and bloodstream infection (6.12%). Recipients with PF positivity and infection had a significantly longer hospital stay compared to those without infection (median of 15 days vs. 11 days; p = 0.001), as well as a longer cold ischemia time (median of 22.7h vs. 19.1h; p = 0.007). No statistically significant differences were observed between the groups regarding age (p = 0.600), acute rejection (8.16% vs. 5.26%; p = 1.000), graft loss (4.08% vs. 5.26%; p = 1.000), or mortality (4.08% vs. 5.26%; p = 1.000). The distribution of underlying renal disease etiologies also did not differ between subgroups, with p-values of 0.702 for diabetes mellitus, 0.232 for hypertension, 0.179 for glomerulonephritis, and 0.664 for other causes.

Among PF-negative recipients, infections were also observed (64%), but these were not microbiologically linked to PF cultures, as expected.

Table 4 compares PF-positive recipients with and without early infections.

**Table 4:** Comparison of clinical characteristics and outcomes of recipients with positive pf cultures, stratified by presence of early infection

Characteristics	PF culture (+) with infection (N = 49)	PF culture (+) without infection (N = 19)	p-value
Age – mean ± SD (years)	46.5 ± 14.2	47.0 ± 11.6	0.6
Etiology of kidney failure – n (%)			
Diabetes mellitus	6 (12.24%)	3 (15.78%)	0.702
Hypertension	16 (32.65%)	3 (15.78%)	0.232
Glomerulonephritis	21 (42.86%)	12 (63.15%)	0.179
Other causes	6 (12.24%)	1 (5.26%)	0.664
Postoperative metrics			
Hospital stay – median (IQR), days	15.0 (11.0–21.5)	11.0 (10.0–14.8)	0.001
Cold ischemia time – median (IQR), h	22.7 (18.1–28.5)	19.1 (16.1–22.0)	0.007
Infections – n (%)			
Urinary tract infection	46 (93.88%)	0	–
Bloodstream infection	3 (6.12%)	0	–
Catheter tip infection	6 (12.24%)	0	–
Secondary outcomes – n (%)			
Acute rejection	4 (8.16%)	1 (5.26%)	1
Graft loss	2 (4.08%)	1 (5.26%)	1
Death	2 (4.08%)	1 (5.26%)	1

Note – Values are presented as mean ± standard deviation, median (interquartile range), or n (%), as appropriate. p-values obtained using chi-square or Mann-Whitney U test, as applicable.

## Discussion

Kidney transplant recipients are at high risk of infection due to several predisposing factors, including preoperative colonization, prolonged hospitalization, extended surgical duration, immunosuppression, and potential contamination of PF used after donor nephrectomy. The effectiveness of prophylactic therapy in preventing infectious complications associated with PF contamination, particularly bacterial contamination, remains uncertain. Although PF collection is not mandatory in all cases^6^, it is routinely performed in transplants from deceased donors.

This retrospective study aimed to assess the PF contamination rate in our center and evaluate its association with early post-transplant infections and clinical outcomes. We found a contamination rate of 27.6%, consistent with a previous systematic review reporting a mean rate of 37%^4^. PF contamination is likely to occur during graft retrieval, as evidenced by the predominance of coagulase-negative staphylococci, which are consistent with donor skin flora. These results are consistent with previous studies^2,7,8,9^. However, targeted antibiotic therapy based on PF isolates did not demonstrate a clear clinical benefit and may contribute to the selection of multidrug-resistant (MDR) organisms^10^.

The agreement between PF isolates and post-transplant infections was 13.23%. Among early urinary tract infection cases, *Klebsiella pneumoniae* was the predominant pathogen, isolated in 66.6% (6/9) of these cases. Each of these episodes occurred in recipients with PF contamination, corresponding to an overall incidence of 8.82% (6/68) among positive PF samples. Our findings are in line with a 2022 Chinese study reporting a 7.3% incidence of donor-derived infections caused by carbapenem-resistant *Klebsiella pneumoniae* ^11^.

The presence of ESKAPE pathogens in PF represents a significant challenge in solid organ transplantation due to their high resistance and association with increased morbidity and mortality^12^. In addition, microorganisms from the intestinal microbiota, such as enterococci and Enterobacteriaceae, were frequently isolated, suggesting possible intra-abdominal contamination during organ procurement.

In the subgroup of recipients with positive PF cultures and early infection, longer hospital stays and prolonged cold ischemia times were observed. However, no significant differences were found regarding acute rejection, graft loss, or mortality, suggesting that these infections, although frequent, were clinically well-managed.

These findings underscore the clinical relevance of systematically evaluating PF culture results in kidney transplantation. Further studies with greater statistical power and multivariate analysis are warranted to better elucidate the clinical impact of positive PF cultures and to develop standardized, evidence-based management strategies aimed at reducing the risk of multidrug-resistant pathogen infections in kidney transplant recipients.

### Limitation

This study has some limitations. First, it was conducted at a single center, which may affect the generalizability of the findings. Second, donor-related risk factors for PF contamination—such as ICU stay duration, concomitant infections, positive donor cultures, and prior antimicrobial use—were not assessed. Furthermore, the absence of antimicrobial susceptibility data limited our ability to interpret microbiological concordance. Lastly, the relatively small sample size may have reduced the power to detect statistically significant associations.

## Conclusion

Preservation fluid contamination is frequently observed in kidney transplant procedures and may be implicated in the development of early infections. While associations with short-term clinical outcomes such as rejection, graft loss, and mortality were not statistically significant, the findings highlight the importance of incorporating standardized microbiological monitoring protocols.

Future multicenter studies with larger sample sizes and comprehensive donor data are warranted to clarify the clinical implications of PF contamination and inform evidence-based prophylactic and therapeutic strategies.

## Data Availability

Additional data supporting the findings of this study may be available by the corresponding author upon reasonable request and subject to appropriate institutional approvals.
