# Supplementary material for: Contamination of perfusion fluid and its impact on kidney transplantation: an observational study from a single Brazilian center
Source: J Bras Nefrol. 2025 Aug 8;47(4):e20250037. doi: 10.1590/2175-8239-JBN-2025-0037en (PMC12334087; doi:10.1590/2175-8239-JBN-2025-0037en)
Supplement: Translation

# Translation: Contaminação do líquido de perfusão e seu impacto no transplante renal: estudo observacional em um centro único brasileiro

**DOI:** 10.1590/2175-8239-JBN-2025-0037pt

**Authors:** Letícia Aguirre Mantoani, Letícia Segura Graciani, Raquel Hernandez Bertine, João Fernando Picollo Oliveira

**Affiliations:** 1 Faculdade de Medicina de São José do Rio Preto, São José do Rio Preto, SP, Brasil.; 2 Faculdade de Medicina de São José do Rio Preto, Departamento de Nefrologia, São José do Rio Preto, SP, Brasil.

**Keywords:** Cultura, Infecções, Perfusão, Transplante

## Abstract

**Introdução:**

Infecções são uma das principais causas de morbimortalidade em receptores de transplante renal. A contaminação do líquido de perfusão (LP) pode constituir uma fonte infecciosa, mas sua relevância clínica permanece controversa.

**Objetivo:**

Avaliar se o LP atua como fonte de infecção precoce (até 30 dias) e sua associação com rejeição aguda, perda do enxerto e mortalidade em até 90 dias.

**Métodos:**

Estudo retrospectivo, observacional e descritivo, baseado em dados de prontuários de pacientes ≥ 18 anos submetidos a transplante renal entre janeiro de 2021 e dezembro de 2023. As variáveis coletadas abrangeram dados demográficos, clínicos e de desfechos pós-transplante.

**Resultados:**

Foram incluídos 246 receptores com dados de cultura do LP; desses, 27,6% (68/246) apresentaram culturas positivas. Cocos Gram-positivos representaram 64,7% dos isolados, bacilos Gram-negativos 35,3% e fungos 2,9%. *Staphylococcus* coagulase-negativo foi detectado em 36,8% das amostras. Houve concordância microbiológica entre o microrganismo isolado no LP e aquele responsável pela infecção precoce em 13,23% (9/68) dos casos, com *Klebsiella pneumoniae* como agente predominante (66,6%). Embora a taxa de infecção tenha sido maior entre os pacientes com LP positivo (72%) em comparação àqueles com LP negativo (64%), a diferença não foi estatisticamente significativa (p = 0,2992). Também não houve associação com óbito (p = 1,000), perda do enxerto (p = 0,8199) ou rejeição aguda (p = 0,5635).

**Conclusão:**

A cultura positiva do LP é frequente e pode estar associada a infecções precoces, ressaltando a importância da vigilância microbiológica.

## Introdução

O transplante de órgãos é considerado o tratamento de escolha para diversas doenças terminais, causadas principalmente pela insuficiência de um órgão. Nesse processo, a preservação do órgão a ser transplantado constitui uma etapa fundamental, permitindo que ele seja retirado do doador e mantido em condições adequadas até sua realocação no receptor. A preservação do enxerto é realizada, principalmente, por meio de um líquido de perfusão (LP), o qual é utilizado em transplantes a partir do momento em que o órgão é removido do doador, de forma a prolongar sua viabilidade fora do corpo humano. É verificado que durante o transplante existe significativa susceptibilidade ao desenvolvimento de infecções devido à contaminação do enxerto por patógenos. A infecção é uma das maiores causas de morbidade e mortalidade após um transplante^1^, podendo ser derivada de patógenos já presentes no doador e que poderão ser identificados no líquido de perfusão.

Um estudo francês relatou que aproximadamente uma em cada cinco amostras de LP de transplante renal apontou culturas positivas^2^, com maior incidência entre doadores falecidos. Fatores potenciais que contribuem para a contaminação do LP incluem a manipulação durante a retirada e transporte do órgão e, principalmente, o manuseio do enxerto durante sua preparação antes da implantação^3^. Uma revisão sistemática e metanálise, publicada em 2018^4^ e baseada em 17 estudos anteriores, relatou uma taxa média de cultura positiva do LP de 37% e uma taxa associada de infecções de 10%.

Embora necessário para manter a viabilidade do órgão, o LP pode atuar como vetor de contaminação entre os processos de retirada do órgão do doador e sua implantação no receptor. Nesse contexto, o presente estudo visa analisar a taxa de contaminação do fluido de perfusão em transplantes renais realizados no Hospital de Base de São José do Rio Preto, bem como verificar sua possível associação com infecções precoces, rejeição do enxerto, perda do enxerto e mortalidade nos primeiros 90 dias após o procedimento.

## Métodos

### Desenho do Estudo e Participante

Trata-se de um estudo retrospectivo, observacional e descritivo, aprovado pelo Comitê de Ética da Faculdade de Medicina de São José do Rio Preto (FAMERP), por meio da Plataforma Brasil (parecer nº 6.561.413; CAAE nº 60072922.3.0000.5415). Todos os dados foram extraídos dos prontuários eletrônicos do hospital.

### Critérios de Inclusão e Exclusão

Foram incluídos pacientes com 18 anos ou mais submetidos a transplante renal entre janeiro de 2021 e dezembro de 2023. Os critérios de exclusão foram: idade inferior a 18 anos ou ausência de dados sobre a cultura do LP. Durante o período do estudo, foram identificados 282 receptores de transplante renal. Desses, 271 tinham idade igual ou superior a 18 anos e 246 apresentavam dados disponíveis da cultura do LP. Assim, 246 pacientes atenderam a todos os critérios de inclusão.

### Estratificação dos grupos para análise

Para fins de análise comparativa, os 246 receptores incluídos foram inicialmente divididos em dois grupos com base no resultado da cultura do líquido de perfusão: grupo LP positivo (n = 68) e grupo LP negativo (n = 178). A partir do grupo LP positivo, foi realizada uma estratificação adicional em dois subgrupos: LP positivo com infecção precoce (n = 49) e LP positivo sem infecção precoce (n = 19), considerando a presença ou ausência de infecção clínica documentada nos primeiros 30 dias após o transplante. Essa subdivisão permitiu avaliar com maior precisão o impacto clínico da positividade da cultura do LP sobre os desfechos clínicos precoces. As comparações estatísticas foram realizadas tanto entre os grupos LP positivo e LP negativo quanto entre os subgrupos LP positivo com e sem infecção.

### Desfechos Primário e Secundário

O desfecho primário deste estudo foi avaliar a incidência de infecção associada à positividade do LP. Os desfechos secundários incluíram a prevalência de culturas positivas, a ocorrência de infecções precoces (até 30 dias após o transplante) e a ocorrência de perda do enxerto, rejeição aguda ou óbito nos primeiros 90 dias após o procedimento.

### Coleta de Dado

Foram coletadas as seguintes variáveis: idade e sexo do paciente, doença renal de base, profilaxia antibiótica, regime de imunossupressão, tempo médio de internação, presença e local da infecção precoce, microrganismos isolados, perda do enxerto, rejeição aguda e óbito. As informações clínicas e cirúrgicas foram registradas de forma anônima em um banco de dados.

### Definiçõe

As culturas do LP foram consideradas positivas na presença de qualquer crescimento microbiano. Considerou-se infecção relacionada ao LP quando o mesmo microrganismo foi posteriormente identificado no receptor. As definições de infecção seguiram os critérios dos *Centers for Disease Control and Prevention* (CDC) e da *National Healthcare Safety Network* (NHSN)^5^. A mortalidade em até 90 dias foi definida como óbito por qualquer causa ocorrido nesse período.

### Protocolos Clínico

Todos os receptores receberam profilaxia intrao-peratória com cefazolina sódica (1 g), mantida por pelo menos três dias. Sulfametoxazol-trimetoprima (400 mg + 80 mg) foi administrada desde a cirurgia por, no mínimo, 90 dias. Alguns pacientes receberam meropenem (500 mg/dia) e/ou vancomicina (15 mg/kg/dia), conforme o nível sérico de vancocinemia, como continuidade de terapêutica antibiótica instituída no doador. A indução imunossupressora foi realizada com basiliximab ou timoglobulina, conforme julgamento clínico. Para a manutenção da imunossupressão, todos os receptores receberam tacrolimus, micofenolato de sódio e prednisona, com ajustes individualizados de acordo com o perfil de risco imunológico.

### Análise Estatística

A análise estatística foi realizada utilizando os programas SPSS para Windows, versão 23.0 (IBM Corp., Armonk, NY, EUA), e GraphPad Instat, versão 3.10 (GraphPad Software, San Diego, CA, EUA). As variáveis categóricas foram apresentadas como frequências absolutas e percentuais, sendo comparadas por meio do teste do qui-quadrado. As variáveis contínuas foram expressas como médias e desvios-padrão ou como medianas e intervalos interquartis (IIQ), conforme a distribuição dos dados. As comparações entre grupos foram realizadas por meio do teste U de Mann-Whitney para variáveis não paramétricas. Todos os testes estatísticos foram bicaudais, e o nível de significância adotado foi de p < 0,05.

## Resultados

Dos 282 receptores de transplante renal identificados no período estudado, 271 tinham 18 anos ou mais, dos quais 246 apresentavam triagens de LP disponíveis no sistema do Hospital de Base. Assim, 36 pacientes foram excluídos da análise. A média de idade foi semelhante entre os grupos: 46,96 ± 13,74 anos no grupo com cultura de LP positiva e 47,46 ± 13,07 anos no grupo com LP negativo (p = 0,7261). A proporção de pacientes do sexo masculino também foi comparável: 64,7% (44/68) no grupo LP positivo e 65,7% (117/178) no grupo LP negativo (p = 0,999).

Todos os pacientes receberam profilaxia antibiótica padrão com cefazolina sódica e sulfametoxazol-trimetoprima. Em uma parcela dos casos, meropenem foi administrado em 2,43% (6/246) dos receptores e/ou vancomicina em 1,62% (4/246). Quanto à imunossupressão, a maioria dos pacientes recebeu indução com basiliximab (78%; 192/246), enquanto 21,9% (54/246) utilizaram timoglobulina.

A Tabela 1 apresenta as características clínicas e demográficas de todos os receptores.

**Tabela 1:** Características clínicas e desfechos de receptores de transplante renal estratificados conforme os resultados da cultura do líquido de perfusão (LP)

Características	Cultura LP negativa (N = 178)	Cultura LP positiva (N = 68)	Valor de p
Sexo, homens – n (%)	117 (65,73%)	44 (64,70%)	0,999
Idade – média ± DP (anos)	47,46 ± 13,07	46,96 ± 13,74	0,7261
Etiologia da falência renal – n (%)			
Diabetes mellitus	12 (6,74%)	10 (14,70%)	0,0877
Hipertensão	40 (22,47%)	19 (27,94%)	0,4644
Glomerulonefrite	50 (28,09%)	32 (47,06%)	0,0076
Outras causas	76 (42,70%)	7 (10,29%)	< 0,0001
Métricas pós-operatórias			
Tempo de internação hospitalar (IIQ), dias	15,0 (11,0–22,0)	14,0 (10,0–20,0)	0,499
Tempo de isquemia fria – mediana (IIQ), h	23,0 (19,38–27,0)	20,07 (17,73–26,42)	0,2109
Infecções – n (%)			
Qualquer infecção	114 (64,0%)	49 (72,0%)	0,2992
Infecção do trato urinário	97 (54,49%)	47 (69,11%)	0,0527
Infecção de corrente sanguínea	11 (6,17%)	3 (4,41%)	0,8199
Infecção do cateter venoso central	23 (12,92%)	6 (8,82%)	0,5027
Desfechos secundários – n (%)			
Rejeição aguda	8 (4,49%)	5 (7,35%)	0,5635
Perda do enxerto	11 (6,17%)	3 (4,41%)	0,8199
Óbito	11 (6,17%)	4 (5,88%)	1,000

Nota – Valores apresentados como média ± desvio padrão, mediana (intervalo interquartil) ou n (%), conforme apropriado. Valores de *p* obtidos por teste do qui-quadrado ou teste de Mann–Whitney U, quando aplicável.

Entre os 246 receptores incluídos, 27,6% (68/246) apresentaram cultura positiva do líquido de perfusão, sendo 66 monomicrobianas e 2 polimicrobianas, contendo bacilos Gram-negativos (*Acinetobacter baumannii*) associados, respectivamente, a *Klebsiella pneumoniae* e *Enterococcus faecalis*. Dentre os isolados, 64,7% (44/68) eram cocos Gram-positivos, 35,3% (24/68) bacilos Gram-negativos e 2,9% (2/68) fungos. O microrganismo mais frequentemente identificado foi *Staphylococcus* coagulase-negativo (CoNS), presente em 36,8% (25/68) das amostras. Patógenos do grupo ESKAPE (*Enterococcus faecium, Staphylococcus aureus, Klebsiella pneumoniae, Acinetobacter baumannii, Pseudomonas aeruginosa* e *Enterobacter* spp.) corresponderam a 36,8% (25/68) dos isolados.

A Tabela 2 apresenta a distribuição dos microrganismos isolados do LP.

**Tabela 2:** Distribuição dos microrganismos isolados das amostras de líquido de preservação (N = 68)

Microrganismos	No. (percentual)
Cocos Gram-positivos	
CoNS	25 (36,76%)
*Enterococcus faecalis*	7 (10,29%)
*Corynebacterium sp*	3 (4,41%)
*Staphylococcus aureus*	1 (1,47%)
Outros	8 (11,7%)
Bacilos Gram-negativos	
*Klebsiella pneumoniae*	10 (14,70%)
*Enterobacter cloacae*	5 (7,35%)
*Pseudomonas aeruginosa*	4 (5,88%)
*Acinetobacter baumannii*	2 (2,94%)
Outros	3 (4,41%)
Fungos	2 (2,94%)

Abreviações – CoNS: coagulase negative *staphylococcus* (*Staphylococcus epidermidis*, *Staphylococcus haemolyticus*, *Staphylococcus hominis*, *Staphylococcus lugdunensis*, *Staphylococcus capitis*, *Staphylococcus warneri*).

No que se refere à ocorrência de infecção nos primeiros 30 dias, 72% dos pacientes com LP positivo desenvolveram infecção, em comparação com 64% daqueles com LP negativo (p = 0,2992), sem associação estatisticamente significativa. Infecções relacionadas a cateter venoso central (p = 0,5027), infecção de corrente sanguínea (p = 0,8199) e infecção do trato urinário (p = 0,0527) também não apresentaram diferença significativa entre os grupos, embora esta última tenha se aproximado da significância. Foram identificados 9 casos de infecção urinária causados por *Klebsiella pneumoniae*, dos quais 6 estavam associados à contaminação do LP.

Outros desfechos secundários, como óbito (p = 1,000), perda do enxerto (p = 0,8199) e rejeição aguda (p = 0,5635), também não mostraram diferenças significativas. Variáveis contínuas, como tempo de internação (p = 0,499), tempo de isquemia fria (p = 0,2109) e idade do receptor (p = 0,999), não apresentaram associação com os resultados das culturas do LP. Por outro lado, foi observada uma associação estatisticamente significativa entre a etiologia da doença renal e o status da cultura do LP. Causas não tradicionais (excluindo diabetes, hipertensão e glomerulonefrite) foram mais comuns entre os pacientes com LP negativo (42,7% vs. 10,3%; p < 0,0001), sugerindo maior prevalência de comorbidades crônicas entre os que apresentaram culturas positivas.

A Tabela 3 apresenta o perfil dos microrganismos isolados do LP com cultura positiva e concordância microbiológica com infecções precoces.

**Tabela 3:** Microrganismos isolados de receptores com cultura de lp positiva e infecções precoces (N = 49)

Microrganismos	Infecção do trato urinário	Infecção na ponta do cateter	Infecção de corrente sanguínea
CoNS	21	6	2
*Enterococcus faecalis*	3	1	0
*Corynebacterium sp*	1	0	0
Outros cocos Gram-positivos	2	1	0
*Klebsiella pneumoniae*	**9**	0	0
*Enterobacter cloacae*	3	0	1
Outros bacilos Gram-negativos	3	0	0
Fungos	2	0	0
Total	44	8	3

Abreviações – CoNS: coagulase negative *staphylococcus* (*Staphylococcus epidermidis*, *Staphylococcus haemolyticus*, *Staphylococcus hominis*, *Staphylococcus lugdunensis*, *Staphylococcus capitis*, *Staphylococcus warneri*). Nota – Esta tabela inclui apenas microrganismos isolados de receptores com cultura de LP positiva que desenvolveram infecções precoces (n = 49). Duas culturas polimicrobianas de LP resultaram em 70 isolados a partir de 68 amostras.

Observou-se correspondência entre o microrganismo isolado no LP e aquele responsável por infecção precoce (até 30 dias após o transplante) em 13,23% dos casos (9/68), sendo *Klebsiella pneumoniae* o agente etiológico predominante, identificado em 66,6% (6/9) desses casos.

Entre os 68 receptores com cultura positiva do líquido de preservação, 72% (49/68) desenvolveram infecção precoce. A infecção do trato urinário foi o tipo mais frequente (93,88%; 46/49), seguida por infecção associada ao cateter venoso central (12,24%) e infecção de corrente sanguínea (6,12%). Pacientes com LP positivo e infecção apresentaram tempo de internação significativamente maior em comparação àqueles sem infecção (mediana de 15 dias vs. 11 dias; p = 0,001), além de maior tempo de isquemia fria (mediana de 22,7h vs. 19,1h; p = 0,007). Não houve diferença estatisticamente significativa entre os grupos quanto à idade (p = 0,600), rejeição aguda (8,16% vs. 5,26%; p = 1,000), perda do enxerto (4,08% vs. 5,26%; p = 1,000) ou óbito (4,08% vs. 5,26%; p = 1,000). A distribuição das etiologias da doença renal também não diferiu entre os subgrupos, com valores de p de 0,702 para diabetes mellitus, 0,232 para hipertensão arterial, 0,179 para glomerulonefrites e 0,664 para outras causas.

A Tabela 4 apresenta as características clínicas e os desfechos dos receptores com cultura positiva do LP, com e sem infecção precoce.

**Tabela 4:** Comparação das características clínicas e desfechos entre receptores com culturas de lp positivas, estratificados pela presença de infecção precoce

Características	Cultura de LP (+) com infecção (N = 49)	Cultura de LP (+) sem infecção (N = 19)	Valor de p
Idade – média ± DP (anos)	46,5 ± 14,2	47,0 ± 11,6	0,6
Etiologia da falência renal – n (%)			
Diabetes mellitus	6 (12,24%)	3 (15,78%)	0,702
Hipertensão	16 (32,65%)	3 (15,78%)	0,232
Glomerulonefrite	21 (42,86%)	12 (63,15%)	0,179
Outras causas	6 (12,24%)	1 (5,26%)	0,664
Métricas pós-operatórias			
Tempo de internação hospitalar (IIQ), dias	15,0 (11,0–21,5)	11,0 (10,0–14,8)	0,001
Tempo de isquemia fria – mediana (IIQ), h	22,7 (18,1–28,5)	19,1 (16,1–22,0)	0,007
Infecções – n (%)			
Infecção do trato urinário	46 (93,88%)	0	–
Infecção de corrente sanguínea	3 (6,12%)	0	–
Infecção do cateter venoso central	6 (12,24%)	0	–
Desfechos secundários – n (%)			
Rejeição aguda	4 (8,16%)	1 (5,26%)	1
Perda do enxerto	2 (4,08%)	1 (5,26%)	1
Óbito	2 (4,08%)	1 (5,26%)	1

Nota – Valores apresentados como média ± desvio padrão, mediana (intervalo interquartil) ou n (%), conforme apropriado. Valores de *p* obtidos por teste do qui-quadrado ou teste de Mann–Whitney U, quando aplicável.

## Discussão

Receptores de transplante renal apresentam elevado risco de infecções, em razão de múltiplos fatores predisponentes, como colonização pré-operatória, tempo prolongado de internação, duração estendida da cirurgia, uso de imunossupressores e possível contaminação do fluido de perfusão utilizado após a nefrectomia do doador. A efetividade da terapia profilática na redução de complicações infecciosas associadas à positividade do LP, especialmente por bactérias, permanece incerta. Embora a coleta do LP não seja obrigatória em todos os casos^6^, sua realização é recomendada em transplantes com doadores falecidos.

Neste estudo retrospectivo, buscou-se avaliar a taxa de contaminação do LP em nosso centro, bem como o impacto das estratégias preventivas adotadas. Observou-se uma taxa de contaminação de 27,6%, valor compatível com o relatado em revisão sistemática prévia^4^, que apontou uma média de contaminação de 37%. A contaminação durante a obtenção do enxerto parece constituir o principal mecanismo envolvido, evidenciado pela predominância de *Staphylococcus* coagulase-negativo nas culturas, microrganismo comum da flora cutânea do doador. Tais resultados são consistentes com estudos anteriores^2,7-9^.

Entretanto, a antibioticoterapia direcionada aos agentes isolados no LP não demonstrou benefício clínico evidente, além de representar um risco conhecido para o surgimento de bactérias multirresistentes (MDR)^10^.

A concordância entre os microrganismos isolados no líquido de preservação e aqueles posteriormente identificados nos receptores com infecção precoce foi de 13,23%. Entre os casos de infecção urinária, *Klebsiella pneumoniae* foi o patógeno predominante, isolado em 66,6% (6/9) desses episódios. Todos os seis casos foram associados à contaminação do LP, correspondendo a uma incidência geral de 8,82% (6/68) entre as amostras com cultura positiva. Esse achado está alinhado com os resultados de um estudo chinês publicado em 2022^11^, que demonstrou uma incidência de 7,3% de *Klebsiella pneumoniae* resistente a carbapenêmicos derivada do doador.

Os seis patógenos do grupo ESKAPE — *Enterococcus faecium*, *Staphylococcus aureus*, *Klebsiella pneumoniae*, *Acinetobacter baumannii*, *Pseudomonas aeruginosa* e *Enterobacter* spp. — representam um desafio clínico significativo devido à sua resistência antimicrobiana em transplantes de órgãos sólidos, incluindo rins, fígado e coração, estando associados a elevadas taxas de morbidade e mortalidade^12^.

Adicionalmente, microrganismos provenientes da microbiota intestinal, como enterococos e enterobactérias, foram frequentemente isolados, o que sugere possível contaminação intra-abdominal durante a captação do órgão.

No subgrupo de receptores com cultura positiva e infecção precoce, observou-se maior tempo de internação e de isquemia fria. No entanto, não houve impacto significativo sobre rejeição aguda, perda do enxerto ou mortalidade, sugerindo que tais infecções, embora frequentes, foram clinicamente controladas de forma eficaz.

Esses achados reforçam a importância de uma avaliação criteriosa da positividade do LP. Novos estudos, com maior poder estatístico e uso de análise multivariada, são necessários para elucidar com mais precisão o impacto clínico da cultura de LP positiva e estabelecer estratégias de manejo padronizadas, baseadas em evidências, que visem à redução do risco de infecções por patógenos multirresistentes em receptores de transplante renal.

### Limitaçõe

Este estudo apresenta algumas limitações, entre as quais se destaca o fato de ter sido conduzido em um único centro e não ter investigado fatores de risco relacionados ao doador renal para a contaminação do líquido de perfusão, como tempo de internação em UTI, presença de infecções concomitantes, culturas positivas e uso prévio de antibioticoterapia. A ausência de dados relacionados aos perfis de suscetibilidade a antibióticos também limita a validade de nossas descobertas sobre concordância microbiológica. Por fim, o tamanho da amostra também é um fator limitante que pode ter impedido a demonstração de significância estatística em algumas análises.

## Conclusão

A contaminação da cultura do líquido de perfusão é um achado frequente no transplante renal. Os resultados deste estudo estão em consonância com estudos prévios que sugerem uma associação entre a positividade do líquido de perfusão antes do transplante e o desenvolvimento de infecções no período pós-operatório, embora essa relação não tenha se mostrado estatisticamente significativa para a maioria dos desfechos clínicos analisados.

Esses achados reforçam a importância de novos estudos voltados ao desenvolvimento de protocolos de vigilância microbiológica custo-efetivos, bem como à padronização de abordagens profiláticas e terapêuticas. As estratégias futuras devem considerar as características dos microrganismos isolados, de modo a aperfeiçoar a prevenção de infecções no contexto do transplante renal.

## Disponibilidade de Dados

Dados adicionais que corroboram os resultados apresentados neste estudo podem ser disponibilizados pelo autor correspondente mediante solicitação razoável, desde que acompanhada das devidas aprovações institucionais.
